# Clinical Tests of Hearing—I

**Published:** 1909-08-07

**Authors:** 


### Otology.

### CLINICAL TESTS OF HEARING—I.

The clinical investigations of the hearing power are analogous to the physical signs to be elicited in the examination of other bodily organs, but are also subjective in that they depend to a great extent on the patient's own sensations and statements. Tests are employed for two main purposes, the quantitative estimation of the amount of hearing power and the qualitative tests designed to distinguish between diseases of different parts of the auditory apparatus.

The accurate estimation of the degree of hearing is a necessary adjunct of the qualitative tests, and is, of course, also necessary as a standard by which to measure the future improvement during any course of treatment. The watch, though commonly used as a convenient test, is a very fallacious one. As the tick of different watches varies very largely, it cannot be used as a record to be compared with others examined by different observers and tested with other watches. But, more important than this, the tick of each watch varies at different times according to the lapse of time from its last winding and according to other indefinite circumstances. Another important objection is that the watch's tick is largely a musical sound, and therefore the hearing power of any patient, as tested by the watch, will vary according to the loss or retention of audition for high or low notes in the scale and be to a great extent independent of the ordinary capacity for hearing a simple noise. It is important, therefore, to employ a simple mechanical means for testing the hearing; the acoumeter produces, not a musical sound, but a small noisy click which is uniform in intensity, not only for the same instrument, but for all acoumeters. It consists of a movable and a fixed rod attached to a vertical bar; the movable part is lifted by the finger to a constant height and falls upon the fixed rod, thus producing the sound, which should be heard by the normal ear at a distance of sixteen yards. In testing, the eyes should be shut and the other ear firmly closed by the patient and the instrument is gradually approached at right angles to the tested ear until it is distinctly heard.

Speech is a most valuable means of testing hearing, because it is for speech that the hearing is most required, but it needs somewhat more care and experience to obtain reliable results. Both whispered and spoken speech can be employed; the former tests rather the hearing for high-pitched notes and the latter the lower part of the scale. Certain precautions must be taken; the patient should not sit near the wall for fear of reflection of the sound, and therefore the room should be a large one; the ear should be turned directly towards the examiner, the other firmly closed and the eyes should be shut. The patient is told to repeat what he hears and should be approached gradually, the distance being noted. To avoid guessing, isolated words should be employed. The examiner must cultivate a uniform low tone of voice; loud speaking is useless, as the vowel-sounds are alone increased thereby and the consonants are overwhelmed. When using the whisper, the examiner should first almost empty the lungs by a deep expiration, as in this way a uniform loudness is obtained and the whistling of the air avoided. This whisper should be heard by a normal ear at 20 to 25 yards.

To examine the hearing power for musical sounds in different parts of the scale, tuning-forks are required. Three forks are sufficient for all ordinary purposes, namely, C 128, $C^2$ 512, and $C^4$ 2048; the figures represent the number of double vibrations which each fork gives per second. The C fork must be provided with clamps to prevent overtones; in choosing these instruments it is also important to be sure that the higher pitched forks sound for a reasonable length of time, at least sixty seconds, for many $C^4$ forks which are sold have far too short a duration of sound. To strike the forks a light hammer, padded like a drum-stick, is very useful and is necessary for the high-pitched instruments. A uniform gentle stroke should be cultivated, for too powerful a stroke gives rise to over-tones, to avoid which, also, the fork should be struck near the junction of the lower and middle third of the prong. The fork must also be held at a uniform distance of about two inches from the ear and always with the prongs either parallel to, or at right angles to, the meatus; in the diagonal position the sound is much weakened by the interference of the sound-waves from the two prongs, a fact which anyone may readily determine by experiment. The patient is instructed to listen attentively and to raise the hand the moment he ceases to hear the vibration. The examiner then carries the fork to his own ear and notes in seconds the duration over which he hears after the patient has ceased to perceive the sound; the result is expressed as *minus* so many seconds from the normal. This is a far more accurate method than to note the length of time during which the patient hears, as it eliminates variations due to the varying strength of stroke. This testing of the air-conduction with tuning forks of different pitch is of great diagnostic value, for in most cases of chronic middle-ear catarrh and especially in otosclerosis the hearing power for the lower tones, C 128, is markedly diminished, whereas in disease of the internal ear the upper notes are most impaired.

Galton's whistle is very useful for examining the upper limit of audition. It is simply a small whistle actuated by a rubber ball; the length of the pipe, and therefore the pitch of the sound, is altered by turning a screw and is registered on a scale marked to tenths of millimetres; a more elaborate and expensive modification, the Galton-Edelmann whistle, is more accurate, but the former is sufficiently reliable for ordinary purposes. It is held a foot from the ear and screwed up until the patient ceases to hear. The whistle is accompanied by a slight blowing sound which must be separately distinguished by the patient. Also it is difficult to block the other ear so that the shrill noise cannot penetrate, and this may be a source of error in testing unilateral cases; it should be noted whether the sound is still heard on closing the affected ear as well.

